# Elevated serum NLRP3 inflammasome level potentially predicts haemorrhagic transformation and unfavourable outcome in acute ischemic stroke patients

**DOI:** 10.1080/07853890.2025.2601437

**Published:** 2025-12-17

**Authors:** Zejing Lin, Qing Li, Xu Zhang, Yinzhou Wang, Xingyong Chen

**Affiliations:** ^a^ Department of Neurology, Shengli Clinical Medical College of Fujian Medical University, Fuzhou University Affiliated Provincial Hospital, Fuzhou, China; ^b^ Department of Neurology, Minda Hospital of Hubei Minzu University, Enshi, PR China; ^c^ Fujian Academy of Medical Science, Fuzhou, China

**Keywords:** Acute ischemic stroke, NLRP3 inflammasome, occludin, haemorrhagic transformation, prognosis

## Abstract

**Objective:**

To assess the diagnostic value of serum NLRP3 inflammasome and occludin levels in predicting hemorrhagic transformation (HT) and functional outcomes in acute ischemic stroke (AIS).

**Methods:**

AIS patients and matched controls were enrolled between June and November 2021. Serum biomarkers were measured and correlated with infarct volume, stroke severity, HT occurrence, and prognosis. Predictive performance was evaluated using logistic regression and ROC curves.

**Results:**

A total of 156 AIS patients and 55 controls were included. AIS patients showed significantly elevated serum NLRP3 (56.59 pg/mL vs. 33.24 pg/mL) and occludin levels (97.42 ng/mL vs. 44.54 ng/mL) (both *p* <0.001). Both biomarkers correlated positively with infarction volume and NIHSS scores. HT occurred in 19.2% of patients, who exhibited markedly higher NLRP3 and occludin levels (both p<0.001). NLRP3 levels differed significantly between HI and PH subtypes (*p* = 0.042). Logistic regression identified infarct volume, creatinine, reperfusion therapy, and NLRP3 as independent predictors of HT. Poor functional outcomes were associated with older age, atrial fibrillation, HT, larger infarcts, higher NIHSS scores, and elevated serum biomarkers (all *p* <0.05). Age, NIHSS, LDL-C, and NLRP3 independently predicted prognosis. The optimal NLRP3 cut-off for predicting HT was 80.86 pg/mL (AUC 0.911), and for poor prognosis 82.75 pg/mL (AUC 0.663). Combining NLRP3 with NIHSS significantly enhanced prognostic accuracy (AUC 0.903).

**Conclusions:**

Elevated serum NLRP3 inflammasome levels represent a promising biomarker for predicting HT and unfavorable outcomes in AIS patients.

## Introduction

Stroke remains a leading cause of mortality and long-term disability, particularly in undeveloped countries [1]. Haemorrhagic transformation (HT) is a common and clinically significant complication of acute ischemic stroke (AIS), occurring either spontaneously or following reperfusion therapies, and is associated with increased morbidity and mortality. Disruption of the blood–brain barrier (BBB) is a key pathological event after stroke and markedly elevates the risk of HT, thereby serving as an important predictor of unfavourable outcomes [2]. Although numerous biomarkers have been investigated for their ability to predict HT, findings across studies remain inconsistent. The substantial heterogeneity of AIS further complicates the identification of reliable indicators that accurately reflect disease progression and prognosis. Blood-derived biomarkers are particularly attractive because they are minimally invasive, rapidly measurable, quantitative and reproducible [3]. Among these, tight junction-associated proteins such as occludin, claudin-5 and ZO-1, which are essential for BBB integrity, have been explored as potential predictors of stroke severity and outcomes [4–6]. Other biomarkers, including neurofilament light chain and occludin, have also shown promise in recent studies [7–9], yet none have reached sufficient consensus for incorporation into routine clinical practice. Given the intricate interplay between BBB disruption, inflammatory responses and ischemic brain injury, we aimed to investigate both structural BBB-related markers (e.g. occludin) and inflammatory markers (e.g. NLRP3 inflammasome) to evaluate their individual and combined prognostic value in AIS.

Immunity and inflammation play central roles in the pathophysiology of ischemic stroke [10]. A broad range of inflammatory mediators, including the immunoproteasome, have been implicated in the development of HT and unfavourable functional outcomes [11,12]. Among these mediators, the NOD-like receptor family pyrin domain-containing 3 (NLRP3) has gained particular attention as a critical driver of stroke-related neurovascular injury [13,14]. Activation of the NLRP3 inflammasome triggers caspase-1-dependent maturation and release of the proinflammatory cytokines interleukin-1β (IL-1β) and IL-18, thereby amplifying neuroinflammation and secondary brain damage [15]. Accumulating evidence indicates that NLRP3 activation exerts deleterious effects in both ischemic and haemorrhagic stroke models [13,14]. Clinical and experimental data further show that risk factors such as hyperglycaemia markedly potentiate NLRP3 inflammasome activity, exacerbating ischemic injury and promoting HT [16,17]. Conversely, pharmacological or genetic inhibition of NLRP3 has been shown to attenuate endothelial hypoxic injury, preserve BBB integrity and improve neurological outcomes in preclinical studies [18,19].

Based on these findings, we hypothesized that circulating NLRP3 inflammasome components, together with occludin, a structural marker reflecting BBB disruption, may serve as meaningful prognostic biomarkers in AIS. In this single-centre prospective study, we therefore sought to evaluate the relationships between plasma NLRP3 inflammasome levels, occludin concentrations, HT and 90-day clinical outcomes in AIS patients, with the goal of determining their individual and complementary prognostic value.

## Research objectives and methods

### Research participants

**Figure 1.**
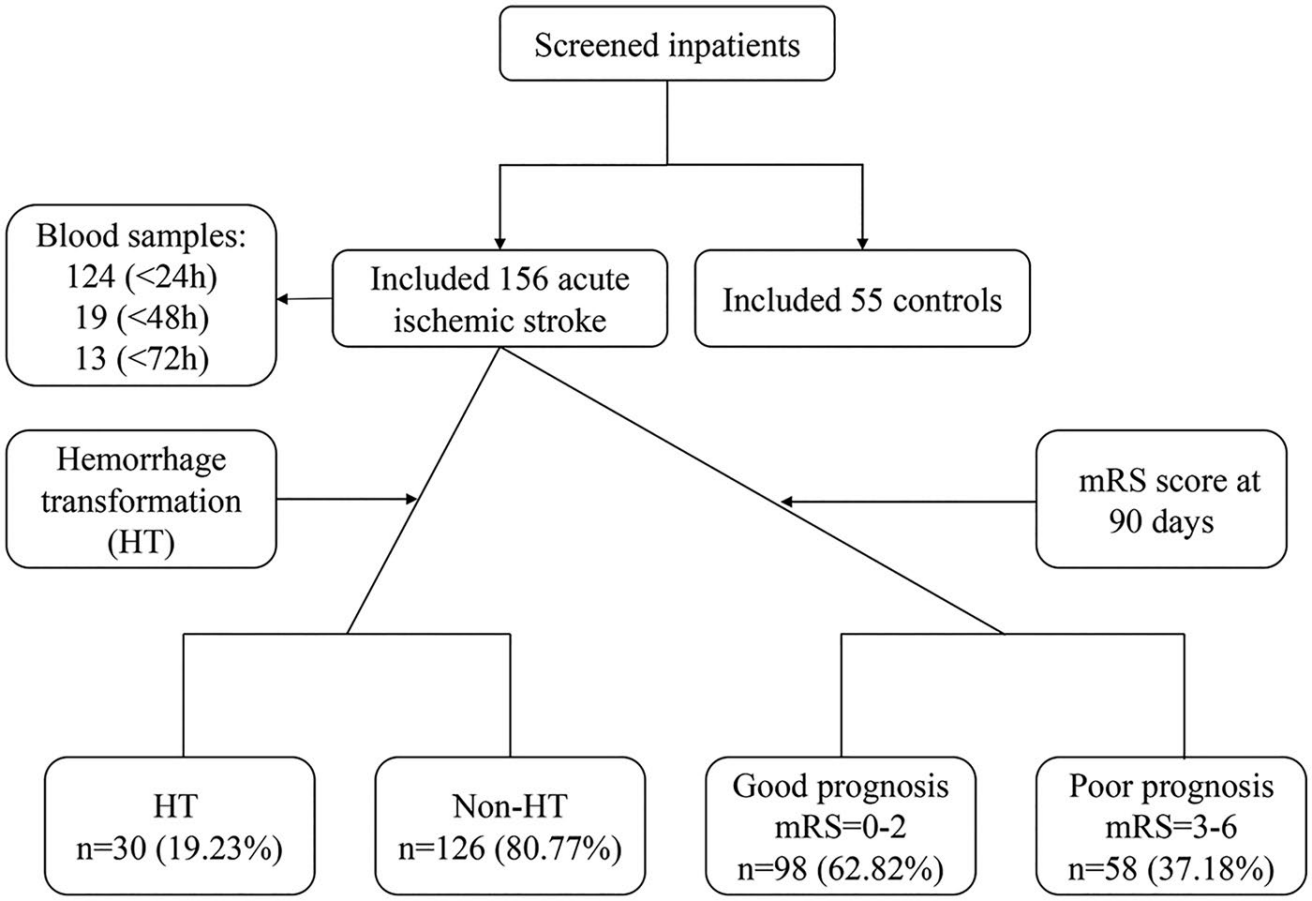
Study flowchart. mRS: modified Rankin Scale.

Patients with AIS and matched controls were prospectively recruited between June 2021 and November 2021 from the Department of Neurology at our hospital. The study flowchart is shown in Figure 1. The study protocol received approval from the Ethics Committee of Fujian Provincial Hospital, Shengli Clinical Medical College of Fujian Medical University (No. K2021-05-045) and written informed consent was obtained from the patient. The inclusion criteria for AIS patients were as follows: (1) age >18 years old; (2) first-ever ischemic stroke with no prior disabilities, and admission occurring within three days of stroke onset; (3) diagnosis of AIS confirmed through clinical assessment and imaging studies, including brain computed tomography (CT) and magnetic resonance imaging (MRI) scans [20]; (4) no history of SARS-CoV-2/COVID-19 infection. Exclusion criteria included: (1) other stroke types such as transient ischemic attack, intracerebral haemorrhage or subarachnoid haemorrhage; (2) presence of inflammatory or infectious diseases, malignancies, haemorrhagic disorders or severe renal and hepatic insufficiency; (3) history of brain tumours, traumatic injury or neuropsychiatric disorders; (4) incomplete clinical or imaging data; (5) patients or their immediate family members or legal guardian unable to give consent to participate in the study. According to our preliminary experimental results, the sample size estimation for AIS group and the healthy control group is 55 cases. In addition, for AIS group, in order to further observe the occurrence of HT and the results of the 3-month follow-up, the sample size for AIS group has been increased. Based on this formula,

$$N={\left(\frac{Z_{1-\alpha /2}\times \sqrt{\rho_{0}\times \left(1-\rho_{0}\right)}+Z_{1-\beta }\times \sqrt{\rho \times \left(1-\rho \right)}}{\delta }\right)}^{2},$$

the sample size was estimated, taking into account a potential dropout rate of 10% during follow-up. Ultimately, a sample size of 156 stroke cases was selected.

The control group participants were prospectively enrolled based on stringent inclusion criteria. These individuals were hospitalized for conditions unrelated to systemic pathophysiological processes, including but not limited to transient dizziness/vertigo, migraine and insomnia. All controls were carefully matched to AIS patients according to age, sex, hypertension status and other cerebrovascular risk factors. Key inclusion criteria for controls included: age >18 years; absence of stroke symptoms or focal neurological deficits; negative neuroimaging findings (CT/MRI) ruling out cerebrovascular events; no history of systemic inflammatory/infectious diseases; malignancies or haematological disorders; severe cardiovascular diseases (including chronic heart failure (NYHA class II–IV), cardiomyopathy or prior myocardial infarction); significant hepatic or renal dysfunction (eGFR <60 mL/min/1.73 m^2^).

### Data collection

Comprehensive clinical data were prospectively recorded by study neurologists, capturing: (1) baseline characteristics (age, sex), (2) comorbidities (hypertension, diabetes mellitus and other relevant conditions), (3) diagnostic workup (laboratory tests, neuroimaging, cerebrovascular/cardiac evaluations) and (4) therapeutic interventions (reperfusion and non-reperfusion approaches). Stroke severity was evaluated using the National Institutes of Health Stroke Scale (NIHSS) scores, with classifications of mild (NIHSS 0–6), moderate (NIHSS 7–15) and severe (NIHSS ≥16) [4]. Stroke etiology was categorized according to the Trial of Org 10172 in Acute Stroke Treatment (TOAST) criteria, including large-artery atherosclerosis, cardioembolic events, small vessel disease and other or unknown causes [21]. Additionally, stroke subtypes were further divided based on the size of the infarction as observed on CT or MRI using the Pullicino formula: infarct volume = Π/6 × *a* × *b* × *c*, where *a* and *b* represent the largest perpendicular diameters measured on imaging and *c* is the slice thickness [22]. Infarction sizes were classified into large infarction group (>10 cm^3^), middle infarction group (4.1–10 cm^3^) and small infarction group (<4 cm^3^) [23]. The occurrence of HT was assessed through follow-up brain CT or MRI scans conducted approximately 5 ± 2 days post-stroke onset or immediately in cases of clinical deterioration. HT subtypes were defined according to ECASS-II criteria: haemorrhagic infarction (HI1/HI2) and parenchymal haemorrhage (PH1/PH2) [24]. All radiographic evaluations were conducted by a neuroradiologist blinded to patient characteristics and treatment assignments. Functional outcomes at 90 days were evaluated using the modified Rankin Scale (mRS), where scores 0–2 defined good outcomes and 3–6 indicated poor outcomes [12].

### Measurement of serum NLRP3 inflammasome and occludin levels

Venous blood samples were collected on admission within three days of onset of AIS (124 patients’ blood samples were collected less than 24 h after stroke onset, 19 less than 48 h, and 13 less than three days after stroke onset). The blood samples collected in EDTA tubes were centrifuged at 3000 × *g* for 20 min to separate the serum. Then, the supernatant was carefully collected and immediately stored at −80 °C until further analysis. Serum levels of the NLRP3 inflammasome and occludin were quantified using enzyme-linked immunosorbent assay (ELISA) kits sourced from Shanghai Meilian Biological Technology Co., Ltd. (Shanghai, China). All assays were conducted following the manufacturer’s instructions. The measurements were carried out by researchers who were blinded to this study. Duplicate well measurements were performed for every sample within a single assay, and the averaged data were recorded.

### Statistical analysis

Statistical analyses were performed using IBM SPSS Statistics (version 28.0; IBM Corp., Armonk, NY). Normally distributed data are presented as mean ± standard deviation (SD) and compared using two independent-sample *t*-tests. Non-normally distributed variables are expressed as median with interquartile ranges (IQRs) and compared using the Mann–Whitney *U*-test or Kruskal–Wallis test, as appropriate. Categorical variables are summarized as counts and percentages and compared using the *χ*^2^ test or Fisher’s exact test. Spearman’s correlation coefficients were calculated to examine the associations between biomarker levels and clinical parameters. Logistic regression analyses were conducted to evaluate predictors of HT and 90-day functional outcomes. For multivariate modelling, the enter method was applied. Importantly, candidate variables were selected *a priori* based on clinical relevance, biological plausibility and supporting evidence from previous literature, rather than solely on the results of univariate comparisons, ensuring a theory-driven modelling framework. All variables deemed clinically essential were retained in the final model irrespective of their univariate *p* values.

Model performance was assessed using receiver operating characteristic (ROC) curve analyses, with the area under the curve (AUC) calculated for each predictor and combined models. Comparisons of AUCs were performed using DeLong’s test. Variance inflation factors (VIFs) were examined to assess multicollinearity. Statistical significance was defined as a two-tailed *p* value <0.05.

## Results

### Clinical characteristics

A total of 156 first-ever AIS patients (99 men, 63.5%) were enrolled in the present study. Meanwhile, 55 controls (aged 65.80 ± 11.60 years; 29 men and 26 women) were finally recruited in the current study. Detailed demographic and clinical characteristics of both groups are summarized in Table 1. Parameters such as age, sex distribution and the prevalence of comorbidities including hypertension, diabetes mellitus and hyperlipidaemia showed no significant differences between the ischemic stroke patients and the control group (all *p* > 0.05). Furthermore, laboratory findings including serum levels of fibrinogen, D-dimer, albumin, alanine aminotransferase, LDL-C, creatinine, uric acid and haemoglobin A1C were comparable between the two groups (all *p* > 0.05).

**Table 1.** Comparison of baseline characteristics of ischemic stroke patients and matched controls.

Variables	AIS (*n* = 156)	Control group (*n* = 55)	*p* Value
Age, years	68.69 ± 11.62	65.80 ± 11.60	0.115
Male, *n* (%)	99 (63.5)	29 (52.7)	0.161
Hypertension, *n* (%)	119 (76.3)	38 (69.1)	0.293
Diabetes mellitus, *n* (%)	54 (34.6)	15 (27.3)	0.318
Atrial fibrillation, *n* (%)	55 (35.3)	12 (21.8)	0.066
Coronary heart disease, *n* (%)	17 (10.9)	5 (9.1)	0.706
Plasma fibrinogen (g/L)	3.32 (2.81–3.62)	3.12 (2.65–3.52)	0.143
D-dimer (mg/L)	0.60 (0.31–1.59)	0.45 (0.34–0.78)	0.111
Fasting plasma glucose (mmol/L)	5.52 (4.53–7.69)	5.41 (4.66–7.94)	0.707
Low-density lipoprotein cholesterol (mmol/L)	2.82 ± 0.90	2.85 ± 0.84	0.819
Creatinine (µmol/L)	74.50 (64.00–85.50)	71.00 (61.50–82.50)	0.270
Uric acid (µmol/L)	339.50 (271.50–387.50)	331.00 (248.50–374.00)	0.104
White blood cell count (×10^9^/L)	7.80 (6.50–10.35)	7.70 (6.90–8.50)	0.228
Neutrophil percentage (%)	70.45 ± 10.72	68.56 ± 7.13	0.145
Platelet count (×10^9^/L)	218.50 (181.00–263.00)	225.00 (196.00–287.00)	0.206
Haemoglobin A1C (%)	6.00 (5.70–7.10)	6.00 (5.60–7.15)	0.433

### Comparison of serum NLRP3 inflammasome and occludin levels between the AIS group and control group

**Figure 2.**
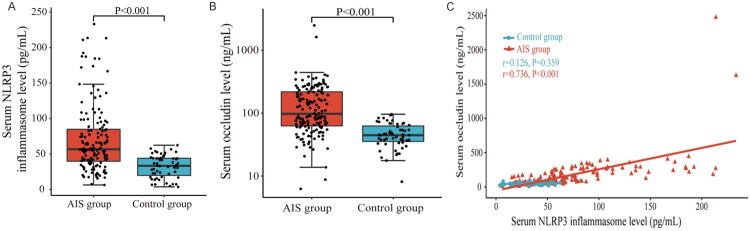
Comparison of serum NLRP3 inflammasome and occludin levels between acute ischemic stroke group and control group. Compared with the control group, serum NLRP3 inflammasome (A) and occludin (B) levels were higher in the AIS group (*p* < 0.001). (C) A strong correlation between serum NLRP3 inflammasome and occludin was found in the AIS group (*r*_s_ = 0.736, *p* < 0.001), but there was no significant correlation between serum NLRP3 inflammasome and occludin in the control group (*p* = 0.359).

Compared to the normal control group, the patients in the AIS group demonstrated significantly elevated serum levels of NLRP3 inflammasome and occludin upon admission (AIS group: NLRP3 inflammasome 56.59 (39.14–84.98) pg/mL vs. control group: NLRP3 inflammasome 33.24 (19.68–43.96) pg/mL, *p* < 0.001, Figure 2A; AIS group: occludin 97.42 (62.25–219.62) ng/mL vs. control group: occludin 44.54 (35.40–62.46) ng/mL, *p* < 0.001, Figure 2B). Moreover, Spearman’s rank correlation coefficient analysis revealed a significant positive correlation between serum NLRP3 inflammasome levels and occludin levels in the AIS group (*r*_s_ = 0.736, *p* < 0.001), while no such correlation was observed in the control group (*p* = 0.359) (Figure 2C).

### Correlation of NLRP3 inflammasome and occludin levels with gender, infarction volumes, stroke etiology and stroke severity

No significant differences were observed in plasma NLRP3 inflammasome and occludin levels between genders (all *p* > 0.05). Table 2 demonstrates that the levels of NLRP3 inflammasome and occludin were highest in the large infarction group (NLRP3 inflammasome: 91.14 (54.72–131.52) pg/mL and occludin: 225.07 (166.25–286.52) ng/mL) among the three groups. A similar pattern was observed across different TOAST subtypes based on stroke etiology. Patients with cardiogenic embolism exhibited the highest concentrations of NLRP3 inflammasome (82.72 (57.88–136.03) pg/mL) and occludin (216.94 (122.92–280.27) ng/mL) among the stroke subtypes (*p* < 0.001). Conversely, the lowest levels of NLRP3 inflammasome and occludin were more commonly found in patients with small artery occlusion and other types of stroke.

**Table 2.** Association between serum NLRP3 inflammasome and occludin levels with gender, infarction volume, TOAST subtypes and stroke severity.

Variables	NLRP3 inflammasome (pg/mL)	*H*-value/*p* value	Occludin (ng/mL)	*H*-value/*p* value
Gender		2345.500/0.080		2330.500/0.071
Male (*n* = 99)	54.56 (37.19–82.83)		83.83 (59.99–194.65)	
Female (*n* = 57)	66.07 (41.68–90.93)		135.04 (68.73–268.76)	
Infarction volume		22.278/<0.001		46.482/<0.001
Large infarction group (*n* = 42)	91.14 (54.72–131.52)		225.07 (166.25–286.52)	
Middle infarction group (*n* = 25)	66.07 (47.88–90.92)		145.39 (75.17–268.16)	
Small infarction group (*n* = 89)	51.18 (35.46–65.63)		71.74 (49.04–107.11)	
TOAST subtypes		25.907/<0.001		46.482/<0.001
Large artery atherosclerosis (*n* = 52)	52.39 (32.29–75.30)		89.26 (59.90–188.43)	
Cardiogenic embolism (*n* = 46)	82.72 (57.88–136.03)		216.94 (122.92–280.27)	
Small artery occlusion (*n* = 51)	50.64 (36.99–66.41)		75.73 (53.77–115.82)	
Other types^a^ (*n* = 7)	50.44 (40.72–54.89)		84.79 (45.24–110.92)	
Stroke severity		25.907/<0.001		27.495/<0.001
Mild group (*n* = 91)	56.34 (37.84–77.17)		83.43 (54.57–161.92)	
Moderate group (*n* = 50)	52.95 (37.17–73.51)		132.59 (65.53–217.66)	
Severe group (*n* = 15)	129.53 (91.14–189.99)		280.27 (211.30–306.57)	

^a^ Other types (including unexplained and other causes).

Upon admission, the NIHSS revealed that there were 91 cases (58.3%) classified as mild, 50 cases (32.1%) classified as moderate, and 15 cases (9.6%) classified as severe. Table 2 demonstrates a significant variation in serum levels of NLRP3 inflammasome and occludin among AIS patients based on stroke severity (*p* < 0.001). Specifically, patients in the severe group had significantly higher serum levels of NLRP3 inflammasome and occludin compared to those in the mild group (NLRP3 inflammasome: 129.53 (91.14–189.99) pg/mL vs. 56.34 (37.84–77.17) pg/mL, *p* < 0.001; occludin: 280.27 (211.30–306.57) ng/mL vs. 83.43 (54.57–161.92) ng/mL, *p* < 0.001).

### Comparison of serum NLRP3 inflammasome and occludin levels between HT group and non-HT group

**Figure 3.**
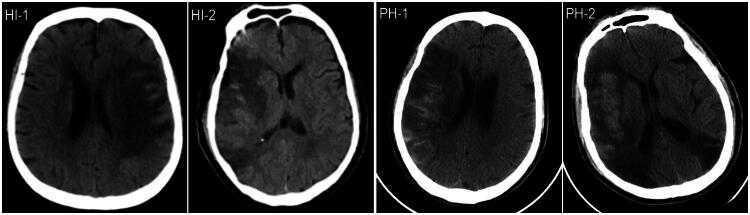
Different types of acute ischemic stroke haemorrhagic transformation confirmed by brain CT.

During hospitalization, 30 patients (19.2%) developed HT. According to the ECASS criteria, these patients were categorized into the following groups: HI1 (*n* = 7, 5%), HI2 (*n* = 10, 6%), PH1 (*n* = 10, 6%) and PH2 (*n* = 3, 2%) (Figure 3). Significant differences were observed in age, percentage of atrial fibrillation and smoking history, TOAST classification, infarct volume classification, admission NIHSS score, D-dimer, creatinine, uric acid, LDL-C, white blood cell count, neutrophil percentage and reperfusion therapy (*p* < 0.05) (Table 3). Notably, the serum levels of NLRP3 inflammasome and occludin were higher in the HT group compared to the non-HT group (Table 3). Due to the small number of patients in each subgroup, HI1 and HI2 were combined into the HI group (*n* = 17, 11%), while PH1 and PH2 were combined into the PH group (*n* = 13, 8%). The study found that the serum NLRP3 inflammasome level was higher in the PH group (172.15 pg/mL, 95% confidence interval (CI) 108.57–184.68) compared to the HI group (98.43 pg/mL, 95%CI 82.26–140.33) (*p* = 0.042). However, there was no significant difference in serum occludin levels between the PH group and the HI group (PH group: 284.24 (255.32–315.63) ng/mL vs. HI group: 259.68 (210.91–297.33) ng/mL; *p* = 0.414).

**Table 3.** Comparison of demographic characteristics, clinical information and biomarker levels between the HT and non-HT groups.

Variables	All (*n* = 156)	Non-HT (*n* = 126)	HT (*n* = 30)	*p* Value
Age, years	68.69 ± 11.62	66.92 ± 11.68	76.10 ± 7.91	<0.001
Male, *n* (%)	99 (63.5)	82 (65.1)	17 (56.7)	0.390
Hypertension, *n* (%)	119 (76.3)	97 (77.0)	22 (73.3)	0.673
Admission systolic blood pressure (mmHg)	150.37 ± 21.06	150.46 ± 21.26	150.00 ± 20.56	0.915
Admission diastolic blood pressure (mmHg)	82.27 ± 13.36	81.98 ± 12.97	83.50 ± 15.08	0.576
Diabetes mellitus, *n* (%)	54 (34.6)	44 (34.9)	10 (33.3)	0.870
Atrial fibrillation, *n* (%)	55 (35.3)	30 (23.8)	25 (83.3)	<0.001
Coronary heart disease, *n* (%)	17 (10.9)	13 (10.3)	4 (13.3)	0.880
TOAST classification, *n* (%)				<0.001
Large artery atherosclerosis	52 (33.3)	46 (36.5)	6 (20.0)	
Cardioembolic	46 (29.5)	22 (17.5)	24 (80.0)	
Small vessel disease	51 (32.7)	51 (40.5)	0 (0)	
Other types^a^	7 (4.5)	7 (5.5)	0 (0)	
Infarct volume, *n* (%)				<0.001
Large infarction	42 (26.9)	19 (15.1)	23 (76.7)	
Medium infarction	25 (16.0)	20 (15.9)	5 (16.7)	
Small infarction	89 (57.1)	87 (69.0)	2 (6.7)	
Admission NIHSS score	5 (2–10)	4 (2–8)	15 (5–17)	<0.001
Plasma fibrinogen (g/L)	3.32 (2.81–3.62)	3.32 (2.81–3.52)	3.34 (2.69–4.44)	0.516
D-dimer (mg/L)	0.60 (0.31–1.59)	0.45 (0.25–1.07)	1.04 (0.68–2.24)	<0.001
Creatinine (µmol/L)	74.50 (64.00–85.50)	72.00 (62.00–83.00)	84.00 (72.00–104.00)	0.002
Uric acid (µmol/L)	339.50 (271.50–387.50)	328.00 (265.00–384.00)	370.50 (313.00–437.00)	0.035
Low-density lipoprotein cholesterol (mmol/L)	2.82 ± 0.90	2.89 ± 0.90	2.52 ± 0.86	0.043
Fasting plasma glucose (mmol/L)	5.52 (4.54–7.64)	5.12 (4.46–7.22)	6.77 (5.51–8.05)	0.013
Glycated haemoglobin (%)	6.00 (5.70–7.10)	6.00 (5.70–7.10)	6.15 (5.70–7.10)	0.780
White blood cell count (×10^9^/L)	7.80 (6.50–10.35)	7.70 (6.20–9.90)	9.45 (7.30–11.70)	<0.001
Neutrophil percentage, *n* (%)	70.45 ± 10.72	68.52 ± 9.96	78.56 ± 10.15	<0.001
Platelet count (×10^9^/L)	218.50 (181.00–263.00)	222.00 (185.00–265.00)	196.50 (163.00–248.00)	0.120
Reperfusion therapy, *n* (%)	31 (19.9)	16 (12.7)	15 (50.0)	<0.001
Statin, *n* (%)	156 (100.0)	126 (100.0)	30 (100.0)	1.000
Antithrombotic therapy, *n* (%)				0.074
Unused	23 (14.7)	15 (11.9)	8 (26.7)	
Antiplatelet therapy	121 (77.6)	102 (81.0)	19 (63.3)	
Anticoagulant therapy	12 (7.7)	9 (7.1)	3 (10.0)	
Neuroprotective agents				
Dibenzoylmethane, *n* (%)	147 (94.2)	120 (95.2)	27 (90.0)	0.269
Edaravone dexborneol, *n* (%)	129 (82.7)	103 (81.7)	26 (86.7)	0.522
Biomarkers levels				
NLRP3 inflammasome (pg/mL)	56.59 (38.63–85.12)	52.15 (34.50–66.74)	121.48 (88.57–173.82)	<0.001
Occludin (ng/mL)	97.42 (61.86–220.59)	81.99 (54.81–152.72)	273.21 (216.22–315.63)	<0.001

^a^ Other types (including unexplained and other causes).

### The usefulness of serum NLRP3 inflammasome and occludin in predicting HT

**Figure 4.**
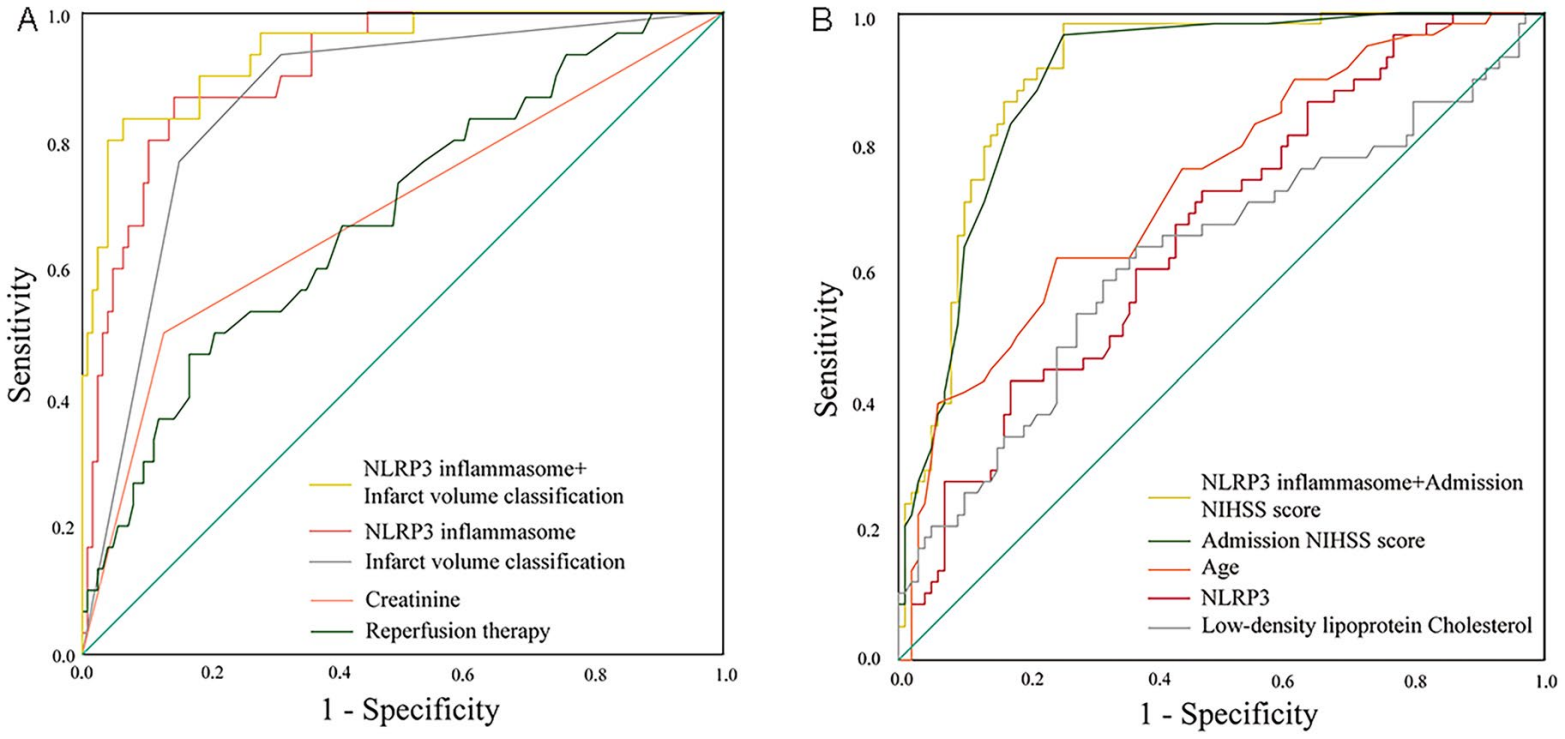
ROC curve of serum NLRP3 inflammasome in predicting haemorrhagic transformation (A) and prognosis (B) in patients with acute ischemia stroke, respectively. (A) AUC value is 0.911 (95%CI: 0.860–0.962, *p* < 0.001), optimal cut-off point value is 80.86 pg/mL, sensitivity is 86.7% and specificity is 85.7%. (B) The AUC value for serum NLRP3 inflammasome predicting poor stroke outcomes was 0.663 (95%CI: 0.577–0.749, *p* = 0.001), with an optimal cut-point value of 82.75 pg/mL (sensitivity 43.1% and specificity 82.7%). Combining elevated serum NLRP3 inflammasome levels with NIHSS scores at admission significantly improved the predictive accuracy for the clinical prognosis of acute cerebral infarction (AUC 0.903, 95%CI: 0.855–0.951, *p* < 0.001; DeLong’s test: AUC difference of 0.240, *Z*-value: 5.054, 95%CI: 0.147–0.333, *p* < 0.001; sensitivity 98.3%, specificity 74.5%).

Univariate analysis (Table 4) revealed significant differences between HT and non-HT groups for multiple factors including age, atrial fibrillation, TOAST classification, infarct volume, admission NIHSS score, creatinine, uric acid, white blood cell count, neutrophil percentage, reperfusion therapy and serum levels of both NLRP3 inflammasome and occludin. All significant variables were subsequently included in multivariate analysis. The final multivariate model (Table 4) demonstrated that infarct volume (adjusted OR 5.53, *p* = 0.025), renal function (per 1 μmol/L creatinine increase: OR 1.09, *p* = 0.015), reperfusion therapy (OR 16.23, *p* = 0.036) and serum NLRP3 inflammasome concentration (per 1 pg/mL increase: OR 1.04, *p* = 0.001) independently predicted HT risk after cerebral infarction. Unexpectedly, these factors including age, atrial fibrillation, TOAST classification, uric acid, white blood cell count, neutrophil percentage and serum levels of occludin, failed to predict HT after infarction (Table 4). Among all evaluated biomarkers, serum NLRP3 inflammasome levels exhibited the strongest association with HT. Notably, the combination of NLRP3 levels with infarct volume showed significantly improved predictive accuracy (Table 5). ROC curve analysis identified an optimal NLRP3 inflammasome cutoff value of 80.86 pg/mL for HT prediction (AUC = 0.911, 95%CI 0.860–0.962), demonstrating high diagnostic accuracy with 86.7% sensitivity and 85.7% specificity (*p* < 0.001; Figure 4A).

**Table 4.** Logistic regression analysis of independent risk factors for haemorrhagic transformation after cerebral infarction.

Variables	Univariate analysis			Multivariate analysis			
	OR	95%CI	*p* Value	OR	95%CI	*p* Value	VIF
Age	1.089	1.041–1.139	<0.001	1.039	0.911–1.185	0.572	1.400
Atrial fibrillation	16.000	5.632–45.451	<0.001	0.300	0.012–7.696	0.467	2.908
TOAST classification^a^	18.909	6.915–51.705	<0.001	9.483	0.727–123.727	0.086	2.672
Infarct volume	6.637	3.438–12.811	<0.001	5.527	1.238–24.685	0.025	1.844
NIHSS score on admission	1.268	1.162–1.383	<0.001	0.986	0.775–1.253	0.906	2.209
Creatinine	1.032	1.013–1.051	0.001	1.087	1.016–1.162	0.015	1.797
Uric acid	1.004	1.000–1.008	0.027	0.999	0.988–1.010	0.804	1.791
White blood cell count	1.133	1.015–1.264	0.026	0.934	0.717–1.218	0.614	1.618
Neutrophil percentage	1.106	1.056–1.158	<0.001	0.990	0.857–1.143	0.890	2.091
Reperfusion therapy	6.875	2.831–16.694	<0.001	16.231	1.202–219.239	0.036	1.263
NLRP3 inflammasome	1.037	1.024–1.051	<0.001	1.044	1.017–1.072	0.001	1.946
Occludin	1.006	1.002–1.009	0.005	1.000	0.996–1.004	0.939	1.642

OR: odds ratio; CI: confidence interval; VIF: variance inflation factor.

^a^ TOAST classification (TOAST classification was divided into cardioembolic and non-cardioembolic, and non-cardioembolic was used as control).

**Table 5.** Related predictors of haemorrhagic transformation after cerebral infarction.

Variables	AUC	95%CI	Sensitivity	Specificity	*p* Value
NLRP3 inflammasome	0.911	0.860–0.962	86.7%	85.7%	<0.001
Infarct volume	0.860	0.790–0.931	93.3%	69.1%	<0.001
Creatinine	0.684	0.577–0.790	80.0%	42.1%	0.002
Reperfusion therapy	0.687	0.569–0.804	50.0%	87.3%	0.002
NLRP3 inflammasome + infarct volume	0.941	0.896–0.985	93.3%	97.6%	<0.001

AUC: area under curve; CI: confidence interval.

### The prognostic role of serum NLRP3 inflammasome and occludin

According to the mRS score, 98 patients (62.82%) were categorized as having a good prognosis, while 58 patients (37.18%) were classified as having a poor prognosis, including seven deaths, after a 90-day follow-up. Table 6 reveals significant differences between the good and poor outcome groups in terms of age, atrial fibrillation, HT, TOAST classification, infarct volume, NIHSS score at admission, fibrinogen, D-dimer, LDL-C, fasting blood glucose, leukocyte count and neutrophil percentage (*p* < 0.05). Additionally, compared to those in the good outcome group, patients in the poor outcome group exhibited higher levels of serum NLRP3 inflammasome (good outcome group: 53.80 (32.93–75.15) pg/mL vs. poor outcome group: 65.44 (48.20–113.26) pg/mL, *p* = 0.001) and occludin (good outcome group: 83.32 (51.45–161.92) ng/mL vs. poor outcome group: 182.38 (76.28–273.86) ng/mL, *p* < 0.001), respectively, compared to those in the good outcome group.

**Table 6.** Demographic and clinical data of AIS patients with good and poor outcomes.

Variables	All (*n* = 156)	Good prognosis (*n* = 98)	Poor prognosis (*n* = 58)	*p* Value
MRS		1(1–2)	4(3–5)	<0.001
Age (years)	68.69 ± 11.62	65.28 ± 11.39	74.45 ± 9.62	<0.001
Male, *n* (%)	99 (63.5)	63 (64.3)	36 (62.1)	0.781
Hypertension, *n* (%)	119 (76.3)	76 (77.6)	43 (74.1)	0.628
Admission systolic blood pressure (mmHg)	150.37 ± 21.06	149.30 ± 20.29	152.19 ± 22.37	0.409
Admission diastolic blood pressure (mmHg)	82.27 ± 13.36	82.22 ± 13.71	82.34 ± 12.88	0.957
Diabetes mellitus, *n* (%)	54 (34.6)	35 (35.7)	19 (32.8)	0.708
Atrial fibrillation, *n* (%)	55 (35.3)	25 (25.5)	30 (51.7)	0.001
Coronary atherosclerotic heart disease, *n* (%)	17 (10.9)	11 (11.2)	6 (10.3)	0.865
Haemorrhagic transformation, *n* (%)	30 (19.2)	12 (12.2)	18 (31.0)	0.004
TOAST classification, *n* (%)				0.016
Large-artery atherosclerosis	52 (33.3)	31 (31.6)	21 (36.2)	
Cardioembolic	46 (29.5)	22 (22.4)	24 (41.4)	
Small vessel disease	51 (32.7)	39 (39.8)	12 (20.7)	
Other types^a^	7 (4.5)	6 (6.1)	1 (1.7)	
Infarct volume classification				<0.001
Large infarction	42 (26.9)	18 (18.4)	24 (41.4)	
Meddle infarction	25 (16.0)	14 (14.3)	11 (19.0)	
Small infarction	89 (57.1)	66 (67.3)	23 (39.7)	
Admission NIHSS score	5 (2–10)	3 (2–5)	10 (7–15)	<0.001
Plasma fibrinogen (g/L)	3.32 (2.811–3.62)	3.32 (2.72– 3.45)	3.36 (2.91–4.11)	0.029
D-dimer (mg/L)	0.60 (0.311–1.59)	0.44 (0.25–1.48)	0.79 (0.36–2.06)	0.026
Creatinine (µmol/L)	74.5 (64.01–85.0)	73.5 (62.0–83.0)	76.0 (65.0–90.0)	0.163
Uric acid (µmol/L)	340 (2721–388)	326 (265 –386)	346 (296 –391)	0.326
Low-density lipoprotein cholesterol (mmol/L)	2.82 ± 0.90	2.66 ± 0.82	3.08 ± 0.98	0.005
Fasting plasma glucose (mmol/L)	5.52 (4.51–7.69)	4.98 (4.37–6.72)	6.54 (5.03–8.11)	0.001
Glycated haemoglobin (%)	6.00 (5.70–7.10)	6.01 (5.70–7.10)	6.00 (5.70–7.10)	0.606
White blood cell count (×10^9^/L)	7.8 (6.51–10.4)	7.2 (6.0–9.3)	9.4 (7.5–11.1)	<0.001
Neutrophil percentage (%)	70.5 ± 10.7	66.6 ± 9.8	77.0 ± 9.0	<0.001
Platelet count (×10^9^/L)	219 (1811–263)	219 (182–260)	216 (177–277)	0.681
Reperfusion therapy, *n* (%)	31 (19.9)	15 (15.3)	16 (27.6)	0.063
Statin, *n* (%)	156 (100.0)	98 (100.0)	58 (100.0)	1.000
Antithrombotic therapy, *n* (%)				0.202
Unused	3 (1.9)	1 (1.0)	2 (3.4)	
Antiplatelet therapy	126 (80.8)	83 (84.7)	43 (74.1)	
Anticoagulant therapy	27 (17.3)	14 (14.3)	13 (22.4)	
Neuroprotective agents				
Dibenzoylmethane, *n* (%)	147 (94.2)	94 (95.9)	53 (91.4)	0.240
Edaravone dexborneol, *n* (%)	129 (82.7)	82 (83.7)	47 (81.0)	0.674
NLRP3 inflammasome (pg/mL)	56.59 (38.63–85.12)	53.80 (32.93–75.15)	65.44 (48.20–113.26)	0.001
Occludin (ng/mL)	97.42 (61.86–220.59)	83.32 (51.45–161.92)	182.38 (76.28–273.86)	<0.001

MRS: modified Rankin Scale.

^a^ Other types (including unexplained and other causes).

Next, the prognostic significance of serum NLRP3 inflammasome and occludin was further investigated using multivariate analysis, incorporating the following variables: age, atrial fibrillation, HT, TOAST classification, infarct volume, NIHSS score on admission, fibrinogen, LDL-C, fasting blood glucose, leukocyte count and neutrophil percentage. The statistical analysis revealed that age (OR 1.130; 95%CI 1.050–1.218; *p* = 0.001), NIHSS score on admission (OR 1.627; 95%CI1.329–1.992; *p* < 0.001), LDL-C (OR 3.196; 95%CI 1.516–6.742; *p* = 0.002) and NLRP3 inflammasome (OR 1.028; 95%CI 1.006–1.051; *p* = 0.012) were independent prognostic factors for poor outcomes in patients with cerebral infarction (Table 7). As depicted in Figure 4B, the AUC value for serum NLRP3 inflammasome predicting poor stroke outcomes was 0.663 (95%CI 0.577–0.749, *p* = 0.001), with an optimal cut-point value of 82.75 pg/mL (sensitivity 43.1% and specificity 82.7%). Surprisingly, serum occludin was not found to be an independent prognostic factor in the multivariate analysis. However, combining elevated serum NLRP3 inflammasome levels with NIHSS scores at admission significantly improved the predictive accuracy for the clinical prognosis of acute cerebral infarction (AUC 0.903, 95%CI 0.855–0.951, *p* < 0.001; DeLong’s test: AUC difference of 0.240, *Z*-value: 5.054, 95%CI 0.147–0.333, *p* < 0.001; sensitivity 98.3%, specificity 74.5%) (Table 8).

**Table 7.** Binary logistic regression analysis of independent risk factors for prognosis of cerebral infarction.

Variables	Univariate analysis		*p* Value	Multivariate analysis			VIF
	OR	95%CI		OR	95%CI	*p* Value	
Age	1.088	1.048–1.129	<0.001	1.130	1.050–1.218	**0.001**	1.421
Atrial fibrillation	3.129	1.574–6.217	0.001	0.713	0.078–6.484	0.764	1.756
Haemorrhagic transformation	3.225	1.419–7.331	0.005	0.134	0.013–1.368	0.090	2.423
TOAST classification			0.021			0.960	1.251
Small vessel disease	Reference						
Large-artery atherosclerosis	2.202	0.939–5.160	0.069	0.661	0.147–2.983	0.591	
Cardioembolic	3.545	1.488–8.445	0.004	0.628	0.049–8.044	0.721	
Other types*	0.542	0.059–4.956	0.587	0.732	0.047–11.304	0.824	
Infarct volume classification	6.637	3.438-12.811	0.001	0.439	0.143–1.343	0.149	2.038
Admission NIHSS score	1.970	1.341–2.893	<0.001	1.627	1.329–1.992	<0.001	2.096
Plasma fibrinogen	1.611	1.094–2.371	0.016	1.409	0.657–3.023	0.378	1.126
Low-density lipoprotein cholesterol	1.713	1.169–2.511	0.006	3.196	1.516–6.742	0.002	1.174
Fasting plasma glucose	1.122	1.006–1.252	0.039	1.069	0.868–1.317	0.529	1.112
White blood cell count	1.215	1.083–1.363	0.001	1.138	0.918–1.411	0.237	1.618
Neutrophil percentage	1.117	1.072–1.164	<0.001	1.064	0.978–1.158	0.149	2.184
NLRP3 inflammasome	1.011	1.004–1.019	0.003	1.028	1.006–1.051	0.012	2.511
Occludin	1.000	0.999–1.002	0.484	0.996	0.992–1.000	0.059	1.665

OR: odds ratio; CI: confidence interval; VIF: variance inflation factor. *Other types: including unexplained and other causes stroke.

**Table 8.** Predictors associated with poor prognosis in cerebral infarction.

Variables	AUC	95%CI	Sensitivity	Specificity	*p* Value
NLRP3 inflammasome	0.663	0.577–0.749	43.1%	82.7%	0.001
NIHSS score on admission	0.895	0.845–0.945	96.6%	74.5%	<0.001
Age	0.734	0.653–0.815	62.1%	75.5%	<0.001
Low-density lipoprotein cholesterol	0.626	0.531–0.721	63.8%	63.3%	0.009
NLRP3 inflammasome + NIHSS score on admission	0.903	0.855–0.951	93.1%	89.8%	<0.001

AUC: area under curve; CI: confidence interval.

## Discussion

Haemorrhagic transformation occurs in approximately 10–40% of patients with AIS and substantially increases morbidity and mortality [25]. However, accurately predicting HT remains clinically challenging, partly due to inconsistent findings regarding biomarker performance across studies. For instance, one investigation reported significantly lower circulating occludin levels in lacunar strokes compared with non-lacunar strokes, with no meaningful association between acute-phase tight junction proteins (occludin, claudin-5, ZO-1) and early outcomes [5], underscoring the need for more robust predictors. In contrast, another study demonstrated markedly elevated serum occludin concentrations in patients with moderate-to-severe cerebral infarction relative to those with milder strokes, and higher occludin levels were associated with poor outcomes in individuals who did not undergo reperfusion therapy [4]. This seemingly paradoxical role of occludin – a core tight junction protein essential for BBB structural stability – formed the rationale for our focused assessment. In our cohort, serum occludin levels were substantially elevated in AIS patients compared with carefully matched controls, with the highest levels observed among patients with large infarctions and severe neurological deficits, as reflected by admission NIHSS scores. These findings align with previous studies [4], suggesting that elevated occludin may reflect both greater structural BBB disruption and increased clinical severity in acute stroke.

Nevertheless, despite its strong univariate associations, occludin did not emerge as an independent predictor of HT or 90-day poor functional outcome in our multivariate analysis, which differs from certain earlier reports. Several factors may explain these discrepancies, including modest sample sizes, heterogeneity in patient populations, and variation in the timing of biomarker sampling. Standardizing these methodological elements may be essential for clarifying the true prognostic utility of occludin in AIS.

Recent studies highlight the pivotal role of the NLRP3 inflammasome in post-stroke neurovascular injury, positioning it as a promising therapeutic target in ischemic stroke [13,26]. Both clinical observations and experimental models consistently demonstrate robust upregulation of NLRP1/NLRP3 inflammasomes and their downstream effector cytokines (IL-1β and IL-18) within peri-infarct regions [19,27]. Genetic deletion of NLRP3 markedly attenuates BBB disruption, diminishes neuroinflammation and substantially improves neurological outcomes following middle cerebral artery occlusion in mice [28]. Similarly, pharmacological inhibition of the NLRP3/caspase-1 pathway reduces infarct volume and preserves BBB integrity across several preclinical stroke models [29,30]. Moreover, NLRP3 activation exacerbates ischemic injury through additional mechanisms, including amplification of the IL-23/IL-17 inflammatory axis [31]. In our study, elevated serum NLRP3 inflammasome levels were not only strongly correlated with circulating occludin but also demonstrated superior predictive performance compared with traditional clinical variables – such as infarct volume, creatinine levels and reperfusion therapy, in identifying patients at risk for HT. These findings underscore the potential value of serum NLRP3 as a clinically informative biomarker and reinforce its relevance to the inflammatory mechanisms driving adverse outcomes in AIS.

Multiple factors influence circulating NLRP3 inflammasome levels. Substantial evidence indicates that hyperglycaemia robustly enhances NLRP3 inflammasome assembly, thereby aggravating HT and worsening clinical outcomes after ischemic stroke [29]. Although we examined the relationship between fasting plasma glucose and NLRP3 concentrations in our cohort, no significant difference was observed between HT and non-HT patients. It is important to note, however, that our study lacked continuous glucose monitoring; thus, future investigations should more comprehensively compare NLRP3 dynamics in AIS patients with and without HT under both hyperglycaemic and normoglycemic states. The molecular mechanisms by which NLRP3 contributes to BBB disruption remain incompletely defined, but current evidence suggests that its effects are multifactorial and highly integrated. Emerging studies identify peroxynitrite, a prototypical reactive nitrogen species, as a potent upstream activator of the NLRP3 inflammasome in intracerebral haemorrhage [32]. This oxidant appears to simultaneously trigger NLRP3 activation and matrix metalloproteinase (MMP) pathways, thereby amplifying BBB breakdown, accelerating HT formation and worsening outcomes in hyperglycaemic stroke patients [29]. Mechanistically, NLRP3-mediated BBB injury is thought to arise through two major pathological routes: (1) amplification of downstream pro-inflammatory signalling cascades and (2) induction of endothelial pyroptosis within the cerebral microvasculature [18]. Growing evidence further indicates that NLRP3 activation perpetuates a vicious cycle of oxidative stress [33] and neuroinflammatory cell infiltration [34], both of which critically enhance BBB permeability. Importantly, these detrimental effects are believed to occur largely through secondary messenger pathways rather than direct structural disruption.

Currently, the prognosis of AIS relies principally on clinical evaluation and neuroimaging findings. However, this paradigm faces substantial limitations due to the inherent subjectivity of symptom assessment and practical constraints in obtaining timely imaging for certain patients. These challenges underscore the critical need for developing objective, readily accessible biomarkers to facilitate early and accurate prognostic stratification in AIS [35]. Growing evidence implicates elevated serum NLRP3 inflammasome levels as a potential predictor for malignant cerebral oedema post-ischemic stroke, though this association requires further validation in large-scale studies to account for potential confounding by stroke severity [36]. Our investigation yielded several key findings: first, we identified significantly higher serum concentrations of both NLRP3 inflammasome and occludin in patients with poor functional outcomes compared to those with favourable recovery. Second, multivariate logistic regression analysis established serum NLRP3 levels as an independent predictor of 90-day poor prognosis (mRS score 3–6), alongside conventional risk factors including advanced age [37], NIHSS score on admission [23] and low-density lipoprotein cholesterol [38]. Most notably, we demonstrated that the combination of serum NLRP3 inflammasome levels with admission NIHSS scores significantly improves prognostic accuracy compared to either parameter alone. This novel composite marker showed superior discriminative capacity for predicting 90-day functional outcomes, as evidenced by ROC curve analysis (AUC 0.903 vs. 0.895 for NIHSS alone, *p* < 0.001). While these results are promising, their clinical applicability warrants verification through multicentre prospective cohort studies with standardized biomarker assessment protocols.

Several limitations should be considered when interpreting our findings. First, the single-centre design with a relatively modest sample size may limit the generalizability of our results and account for the observed discrepancies with prior studies reporting associations between admission D-dimer levels and edaravone dexborneol to stroke prognosis [39,40], our findings did not replicate these associations. Therefore, larger and multicentre studies with diverse cohorts are needed to validate our findings. While the NLRP3 inflammasome’s biological role in cleaving caspase-1 and converting pro-IL-1β/pro-IL-18 to their active forms (IL-1β and IL-18) is well-established, we were unable to measure these downstream inflammatory mediators. This represents a significant knowledge gap, as simultaneous quantification of these cytokines could have provided mechanistic insights into the observed associations between NLRP3 levels and clinical outcomes. Future studies should employ multiplex cytokine profiling to fully characterize the inflammasome activation cascade in acute stroke. In addition, the odds ratios for NLRP3 in the current study were consistently very small which is somewhat unexpected. The causes may be attributed to several factors including biological plausibility, measurement characteristics and temporal dynamics. For example, as a circulating inflammatory mediator, NLRP3 may exert its pathological effects through cumulative exposure rather than threshold-dependent mechanisms, resulting in smaller but clinically relevant effect sizes. Our study design lacked serial measurements of serum NLRP3 inflammasome and occludin levels throughout different phases of AIS progression. This represents a critical methodological gap, given that the dynamic pathophysiological cascades in AIS-including the evolving inflammatory response and BBB disruption-exhibit distinct temporal patterns that may require stage-specific therapeutic interventions. Therefore, expanding the sample size, conducting multicentre studies and performing stratified analyses are essential to enhance the reliability and accuracy of our conclusions.

In conclusion, our findings indicate that serum levels of the NLRP3 inflammasome are significant predictors of HT and adverse outcomes in AIS patients. Regular monitoring of NLRP3 inflammasome levels could enhance risk assessment and enable targeted therapeutic strategies in this population.

## Data Availability

The datasets used and analysed during the current study are available from the corresponding author Xingyong Chen upon reasonable request.
